# Unchanged Faces of Acute Appendicitis: Exploring Presentation and Treatment Amidst the COVID-19 Pandemic

**DOI:** 10.7759/cureus.60674

**Published:** 2024-05-20

**Authors:** Akshay Bavikatte, Sanad Isswiasi, Kyrllos Farag

**Affiliations:** 1 General and Colorectal Surgery, West Suffolk Hospital NHS Trust, Bury St. Edmunds, GBR; 2 General Surgery, West Suffolk Hospital NHS Trust, Bury St. Edmunds, GBR

**Keywords:** complicated acute appendicitis, world pandemic, lenght of hospitalization, covid-2019, diagnosis of acute appendicitis

## Abstract

Introduction

The emergence of the COVID-19 pandemic necessitated the implementation of novel guidelines for managing appendicitis, prompting an evaluation of its effects on patient presentation and treatment at a district general hospital. Healthcare facilities worldwide have adapted protocols to meet the unique challenges of the pandemic, ensuring safe and efficient care. Our study assesses the pandemic's influence on patient demographics, clinical outcomes, surgical procedures, and adherence to guidelines among individuals undergoing emergency appendicitis surgery. Through this investigation, we aimed to determine whether significant deviations occurred in managing acute appendicitis amidst the pandemic.

Methodology

Consecutive adult patients (≥18 years) diagnosed with acute appendicitis were included in two cohorts for this retrospective analysis, comparing cases treated during the COVID-19 pandemic period (April to September 2020) with those treated one year prior. All patients underwent standardized assessments upon emergency department admission, including imaging studies and COVID-19 testing. Demographics, laboratory results, surgical details, and outcomes were compared between the pre- and post-pandemic groups, focusing on their overall management.

Results

The research involved a total of 172 individuals. During the pandemic (April to September 2020), 91 of these participants underwent surgery, which is more than the 81 individuals who had surgery during the same period the previous year (April to September 2019). Preoperative C-reactive protein levels were significantly higher in the pandemic group (*P* = 0.0455). The time from admission to surgery was shorter in the pandemic group (7.5 ± 4.6 vs. 5.8 ± 4.9; *P* = 0.0155). The overall operative and laparoscopic operative times were longer in the pandemic group (65 vs. 71 minutes, *P* = 0.391, and 55 vs. 62 minutes, *P* = 0.1424, respectively). However, these differences were not statistically significant. The number of patients presenting with complicated appendicitis was significantly higher in the pandemic group than in the nonpandemic group (44.4% vs. 61.4%; *P *= 0.034). The length of stay was shorter in the pandemic group (*P* = 0.53).

Conclusions

Our study suggests that surgery for acute appendicitis remains safe and feasible during the COVID-19 pandemic, with comparable outcomes. However, we noted an increase in the number of patients presenting with complicated appendicitis, possibly influenced by national pandemic guidelines in the United Kingdom. Despite this trend, our findings affirm the continued effectiveness of surgical management for acute appendicitis during the pandemic, highlighting the adaptability of healthcare systems in addressing emergent medical needs under challenging circumstances.

## Introduction

In March 2020, the World Health Organization officially declared COVID-19 a global pandemic, prompting rigorous measures worldwide to contain the spread of SARS-CoV-2 and prevent healthcare systems, including the National Health Service (NHS), from becoming overwhelmed [1]. These measures were essential for effective pandemic management and easing the burden on healthcare resources [2].

Simultaneously, it is crucial to maintain consistency in addressing critical surgical emergencies, such as acute appendicitis, which ranks among the most prevalent urgent surgical conditions [3]. Despite the formidable challenges presented by the pandemic, ensuring swift and efficient treatment for acute appendicitis remains a top priority [4].

In light of the changing circumstances, the government introduced several measures, including the restructuring of surgical services across healthcare establishments in the United Kingdom [5]. At our institution, strategic modifications were enacted to efficiently allocate resources and enhance the handling of emergency abdominal and trauma cases, while elective surgeries for non-urgent conditions were temporarily postponed during the height of the pandemic.

Given these circumstances, our retrospective study aimed to evaluate the impact of the implemented measures on the surgical management of acute appendicitis at our institution. Through this analysis, we aim to ascertain the efficacy of our response to the pandemic and identify any departures from standard protocols in managing appendicitis as a surgical emergency during this unprecedented period.

## Materials and methods

A retrospective analysis was conducted on 172 patients who underwent surgery for acute appendicitis at the West Suffolk Hospital. The study included patients who underwent surgery during the pandemic period from April to September 2020 and those who underwent surgery during the same period one year before the implementation of COVID-19 measures. This retrospective study conducted for this research received ethical approval and was registered with the West Suffolk Hospital Audit Committee. Ethical clearance was obtained to ensure compliance with relevant guidelines and regulations. Verbal consent was obtained from all patients included in the study, as all patient identification parameters were anonymized to protect confidentiality and privacy.

Study population

Adult patients aged 18 years or older diagnosed with acute appendicitis were enrolled in two separate groups. Group 1 was the pre-pandemic cohort from April to September 2019, and Group 2 was the pandemic cohort from April to September 2020. Patients were identified by searching electronic patient records using specific International Classification of Diseases, 10th Revision (ICD-10) codes related to appendicitis, acute abdominal pain, or intra-abdominal abscesses. Accident and emergency (A&E) and surgery lists from the relevant period were also reviewed. Patients were included if they were ultimately diagnosed with acute appendicitis. Although no formal calculation was conducted to determine the sample size, a predetermined inclusion period was established.

In both periods, the patients were admitted to the emergency department and subjected to a standardized assessment protocol for acute appendicitis. This protocol comprised extensive history taking, detailed physical examination, routine blood tests, and imaging studies, such as ultrasound or computed tomography (CT) scans, to gauge the severity of appendicitis. During the pandemic, all patients underwent CT scans of the chest and abdomen rather than ultrasound, per the national guidelines.

After admission, all patients underwent real-time polymerase chain reaction (RT-PCR) testing for COVID-19. It is important to note that the time required for RT-PCR analysis did not delay initiating surgical treatment for acute appendicitis. This ensured that patients received prompt and appropriate care regardless of their COVID-19 status, thereby minimizing any potential impact on treatment timelines.

The analysis assessed patient characteristics, including gender and age, along with American Society of Anesthesiology (ASA) scores, laboratory findings such as white blood cell count and C-reactive protein (CRP) levels, duration from admission to surgery, surgical approach (open, laparoscopic, or converted), operative duration, intraoperative observations, conversion frequency, and hospital stay duration between the pre-pandemic and pandemic patient cohorts. Furthermore, the incidence of complicated appendicitis cases was also investigated.

Definition

The severity of appendicitis was assessed based on surgical notes and imaging reports for cases managed conservatively. Uncomplicated appendicitis was characterized by appendix inflammation without signs of necrosis or perforation, as indicated by the surgeon. Complicated appendicitis was defined by appendix inflammation accompanied by gangrene, apparent necrosis, perforation, and/or the presence of abscess formation or perforation, as described by the surgeon and radiologist. Conservative treatment initially involved antibiotics and/or percutaneous drainage of appendiceal abscesses. For patients undergoing surgery, in-hospital delay or time to operation was defined as the duration from the emergency department (ED) presentation to the commencement of surgery.

Statistical analysis

The statistical analysis was performed using STATISTICA version 12.0 for Windows. Descriptive statistical techniques were used for data representation, including mean, standard deviation (SD), median, and percentage. The normality of parameters was assessed using the Kolmogorov-Smirnov test. For numerical parameters, comparisons between two independent means were conducted using Student's t-tests and the Mann-Whitney U test, while categorical data analysis depended on sample size and employed chi-square and Fisher's exact tests. Statistical significance was defined as *P* < 0.05.

## Results

**Figure 1:**
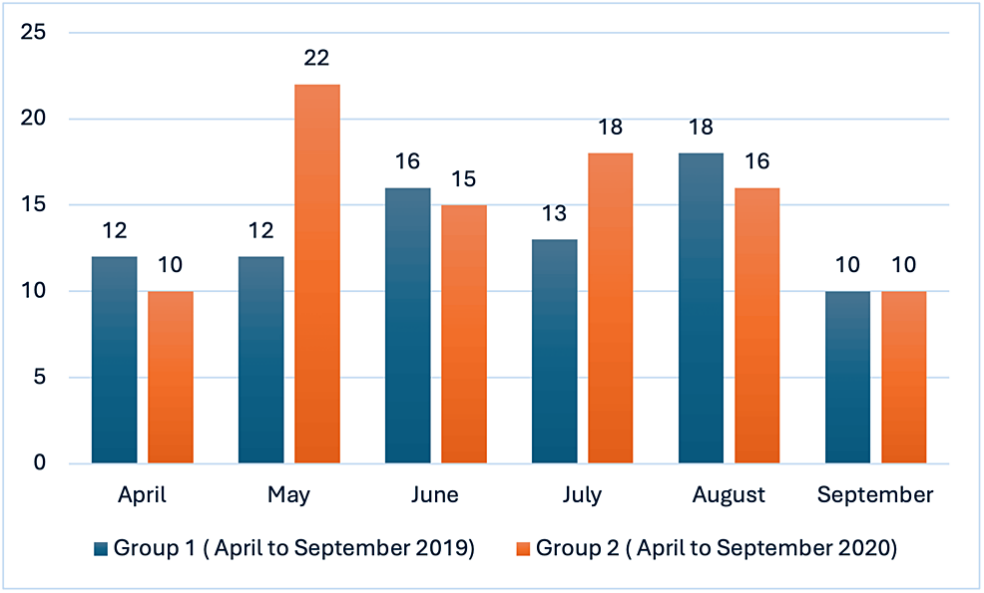
Monthly distribution of appendectomies between the two groups. Group 1: April to September 2019; Group 2: April to September 2020 , The data has been represented in numbers.

A total of 107 male and 55 female patients, comprising 63.8% and 36.2% of the study population, respectively, were operated on with a preoperative diagnosis of acute appendicitis during the study period. A total of 81 patients underwent appendectomies during the pre-pandemic period (Group 1), whereas 91 patients underwent the procedure during the pandemic period (Group 2). The monthly distribution of appendectomies is as follows: in April, 12 (14.8%) in Group 1 and 10 (11.0%) in Group 2; in May, 12 (14.8%) in Group 1 and 22 (24.2%) in Group 2; in June, 16 (19.8%) in Group 1 and 15 (16.5%) in Group 2; in July, 13 (16.0%) in Group 1 and 18 (19.8%) in Group 2; in August, 18 (22.2%) in Group 1 and 16 (17.6%) in Group 2; and in September, 10 (12.3%) in Group 1 and 10 (11.0%) in Group 2, as illustrated in Figure 1.

The median age of males was 34.6 (range 6-83) years and that of females was 31.9 (range 7-76) years. They were statistically nonsignificant; male predominance was seen in both groups (Table 1).

**Table 1:** Patient demographics. *P*-value calculated using the Student's t-test. Data were represented as n, %. Mean ± SD, *P* < 0.05 is considered significant. SD, standard deviation; *n*, number

Characteristics	Group 1 (April to September 2019)	Group 2 (April to September 2020)	*P*-value
Number of patients	81	91	-
Age, years (Mean ± SD)	35.50 ± 17.49	32.13 ± 16.86	0.2288
Age range (years)	8-83	6-75	-
Male (*n*, %)	57 (66.2%)	60 (61.7%)	0.5673

The analysis of preoperative factors revealed several noteworthy findings between the two groups. First, the ASA score, which assesses a patient's overall health status and comorbidities, was similar in both the pre-pandemic and pandemic groups. This suggests that the overall health status and comorbidity burden of patients undergoing appendectomy did not differ significantly between the two periods. Similarly, preoperative white cell count, which can indicate the presence of infection or inflammation, was comparable between the two groups, indicating that the severity of systemic inflammation at the time of presentation was similar.

However, significant differences were observed in other preoperative parameters. Specifically, CRP levels were statistically higher in the pandemic group compared to the pre-pandemic group (97.08 ± 95.54 vs. 64.38 ± 70.10; *P* = 0.0455). Elevated CRP levels indicate systemic inflammation and are commonly used as an acute infection or inflammation marker. The higher CRP levels in the pandemic group may suggest a more severe inflammatory response or a higher burden of infection among patients presenting during the pandemic period.

Furthermore, the time from admission to surgery was statistically shorter for patients in the pandemic group compared to the pre-pandemic group (mean ± SD, 8.3 ± 5.1 [Median 6] vs. 5.2 ± 4.3 [Median 5]; *P* = 0.0155). This indicates that patients in the pandemic group underwent surgery more rapidly after admission than those in the pre-pandemic group. The shorter time to surgery may reflect changes in clinical practice or hospital protocols implemented during the pandemic to prioritize and expedite surgical interventions. The preoperative data have been illustrated in Table 2.

**Table 2:** Preoperative data between the two groups. *P*-value calculation: categorical variables, two-sample t-test; continuous variables, Mann-Whitney U test. The data are represented as *n*, mean ± SD; median. *P* < 0.05 is considered significant. CRP, C-reactive protein; *n*, number; SD, standard deviation

Parameters	Group 1 (*n* = 81) (April to September 2019)	Group 2 (*n* = 91) (April to September 2020)	*P*-value
ASA score (*n*)			
1	41	52	0.4979
2	31	33	
3	9	6	
CRP (mg/L) (Mean ± SD)	63.38 ± 70.10 (Median 32.2)	98.76 ± 94.78 (median 58.1)	0.0455
White cell count, x10^9^/L (Mean ± SD)	14.57 ± 4.15	15.78 ± 5.79	0.1424
Time from admission to surgery (hours), mean±​​​​SD (median)	8.3 ± 5.1 (Median 6)	5.2 ± 4.3 (Median 5)	0.0155

**Figure 2:**
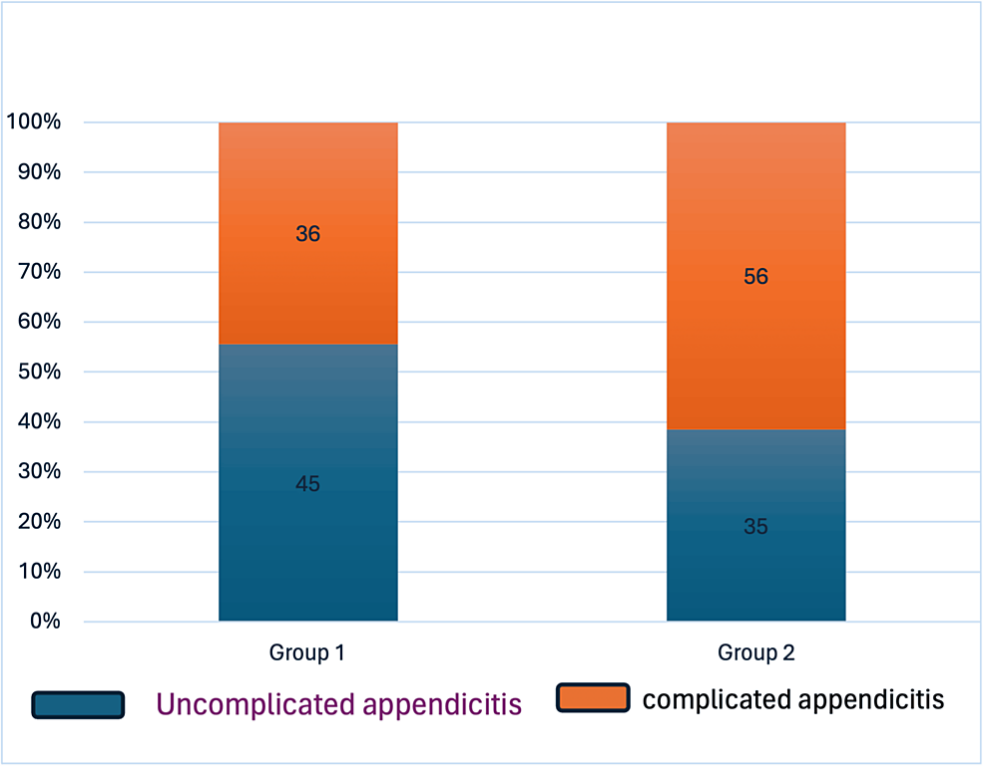
Difference in the presentation of complicated appendicitis between the two groups. Group 1: April 2019 to September 2019; Group 2: April 2020 to September 2020.

The analysis indicates that there was no statistically significant difference in operative times between the two groups overall. Even when looking specifically at laparoscopic procedures, which are often expected to have shorter operative times, no statistically significant difference was observed (69.92 minutes for Group 1 vs. 71.0 minutes for Group 2; *P* = 0.391). This suggests that, on average, the time taken for surgery did not differ significantly between the two groups, regardless of the surgical approach used. Despite similar operative times, there was a notable disparity in the rates of complicated appendicitis between the two groups. Complicated appendicitis was present in 42.25% of cases in Group 1 compared to 56 (61.4%) in Group 2, with this difference being statistically significant. This finding suggests that patients in Group 2 were more likely to present with complicated appendicitis compared to those in Group 1. However, despite this difference in the severity of appendicitis presentation, no statistically significant difference was observed in the length of hospital stay between the two groups. This indicates that while the severity of appendicitis may vary between the groups, it does not necessarily translate into differences in the postoperative recovery period or hospital stay duration. The operative parameters are illustrated in Table 3, and the difference between the complicated appendicitis presentation is shown in Figure 2.

**Table 3:** Operative data between the two groups. *P*-value was calculated using the chi-square test. The data were represented as *n*, mean ± SD; median. *P* < 0.05 is considered significant. SD, standard deviation; *n*, number

Parameters	Group 1 (*n* = 81) (April to September 2019)	Group 2 (*n* = 91) (April to September 2020)	*P*-value
Type of surgery			
Laparoscopic, *n* (%)	65 (80.2%)	73 (80.3%)	0.975
Open, *n* (%)	13 (16.04%)	16 (17.5%)	
Converted, *n* (%)	3 (3.07%)	2 (2.2%)	
Operative time/Minutes (all procedures)	69.92 ± 23.36 (median 65)	71.4 ± 23.3 (median 71)	0.391
Operative time (Laparoscopy)	61.12 ± 24.02 (median 55)	72.01 ± 19.36 (median 62)	0.1424
Operative time (Open procedure)	75.3 ± 23.22 (median 76)	76.92 ± 19.81 (median 77.5)	0.39
Complicated appendicitis rate, *n* (%)	36 (44.4%)	56 (61.4%)	0.034
Length of stay/Days (mean ± SD)	4.8 ± 1.78	4.1± 1.85	0.53

## Discussion

Studies have documented a notable decline in the number of patients presenting with acute appendicitis during the year 2020, with the lowest admissions recorded in April of that year. Emergency admissions for acute appendicitis were reported to have decreased by anywhere from 1.75% to 84% [6]. Additionally, Tankel et al.'s research highlighted a reduced incidence of acute appendicitis by 40.7% in a retrospective cohort analysis spanning seven weeks [7]. The primary factor attributed to the decrease in patient visits to emergency departments is the apprehension surrounding potential SARS-CoV-2 infection in outpatient medical facilities during the COVID-19 pandemic [8].

To support emergency surgical patients effectively and efficiently while also optimizing patient care resources, several countries issued guidelines for triaging emergency surgery patients during the pandemic [9]. Specifically regarding the treatment of uncomplicated appendicitis, these guidelines recommended that surgeons initially employ antibiotics as the primary treatment, reserving appendectomy for cases where symptoms worsen or recur [10]. For cases of complicated appendicitis, the recommendation included the option of intravenous antibiotic therapy, while patients with periappendicular abscesses and perforations were advised to undergo percutaneous drainage or surgery [11].

The research conducted by Orthopoulos et al. offered compelling evidence supporting the notion of a significant uptick in appendicitis-related admissions and appendectomies during the COVID-19 pandemic era. Our study indicated a notable 13.1% increase in such admissions compared to the corresponding period in the previous year and also sheds light on a concurrent rise in the incidence of complicated appendicitis cases [12].

The existing literature underscores a notable increase in WBC and CRP levels among patients diagnosed with acute appendicitis during the COVID-19 pandemic compared to pre-pandemic periods [13]. This observation suggests a correlation between the pandemic and elevated inflammatory markers in individuals presenting with acute appendicitis. Specifically, the study conducted by Ramu et al. accentuated the significance of CRP levels in diagnosing acute appendicitis, particularly in distinguishing between various histopathological findings encountered during appendectomy procedures [14]. In alignment with these findings, our study also contributes to this body of evidence by demonstrating significantly higher preoperative CRP levels during the pandemic. This consistency with existing literature highlights the broader trend of heightened inflammatory responses observed in patients with acute appendicitis amidst the backdrop of the COVID-19 pandemic.

Lotan et al. observed a slightly prolonged duration of hospitalization during the COVID-19 pandemic compared to the period preceding it (12.2 days vs. 9.9 days, *P* = 0.27), along with a marginally extended interval to surgery (6.25 days vs. 5.3 days, *P* = 0.55) [15]. Conversely, Fisher et al. found that COVID-19-positive patients experienced a delayed time from admission to surgery during the pandemic (2.7 days vs. 1.2 days, *P* = 0.034), indicating challenges in surgical case management during this period [16]. Given the heightened frequency of admissions and the increased volume of overall surgical procedures during the COVID-19 pandemic at our trust, elective benign procedures were postponed. This adjustment facilitated effective post-admission organization and patient preparation for surgery. Consequently, there was a notable reduction in the time from admission to surgery compared to the pre-pandemic period, indicative of enhanced efficiency in patient care and surgical management strategies.

During the early stages of the pandemic, UK Surgical Royal Colleges recommended conservative management with antibiotics as the first-line treatment for acute uncomplicated appendicitis, aiming to minimize aerosol generation. In cases where surgery was necessary, open surgery was favored over laparoscopic surgery [17]. Waldman et al. noted a higher frequency of laparoscopic appendectomies during the COVID-19 pandemic compared to the previous year, indicating a continued trend favoring laparoscopic over open appendectomy as the standard of care [18]. However, our study did not identify a significant statistical difference in terms of laparoscopic surgery between the pandemic and pre-pandemic periods. Contrary to the belief that there was a significant increase in laparoscopic appendectomy rates during the pandemic, our findings suggest that the frequency of laparoscopic appendectomies remained relatively stable compared to the pre-pandemic period.

Scheijmans et al. observed a greater proportion of complicated appendicitis cases during the COVID-19 pandemic compared to the previous year (46.9% vs. 38.5%, *P* = 0.003), suggesting an escalation in the severity of appendicitis cases during this period. This finding implies that delayed healthcare-seeking behaviors, possibly influenced by pandemic-related guidance, might have contributed to the increased incidence of complicated appendicitis cases [19]. Similarly, Burgard et al. underscored a significant rise in complicated appendicitis cases attributed to delayed patient presentation during the COVID-19 pandemic. Their study documented a higher proportion of complicated appendicitis cases during the pandemic period, likely stemming from patients delaying consultation with the emergency department upon experiencing symptoms [20]. In alignment with these observations, our study revealed a statistically significant increase in complicated appendicitis presentations during the pandemic compared to preceding years. This collective evidence suggests a concerning trend of delayed healthcare seeking and subsequent exacerbation of appendicitis severity amidst the challenges posed by the COVID-19 pandemic.

The duration of operative time during the pandemic era has yielded varying findings in recent studies. Zhou and Cen's investigation, spanning 2019 and 2020, noted no difference in operative time during the same period [21]. Conversely, Ho et al.'s study indicated a trend toward longer operative times during the pandemic, although this increase did not reach statistical significance. However, their research highlighted a statistically significant extension in operative time during the COVID-19 pandemic [22]. However, our analysis found no statistical disparity in operative time between the two cohorts under examination. These disparate findings underscored the complexity of assessing operative time trends amidst the dynamic circumstances of the COVID-19 pandemic, reflecting the interplay of various factors such as procedural adjustments, resource allocation, and surgical practice adaptations during this challenging period. Likewise, there was no difference between the two groups regarding length of stay within the hospital.

The study's outcomes should be considered with some limitations in mind. Initially, data were collected retrospectively and were only available for individuals who sought treatment at the hospital. Consequently, the proportion of patients with complicated appendicitis in relation to the overall number of individuals with acute appendicitis was likely skewed by the number of patients with mild appendicitis who did not seek hospital treatment and instead experienced the resolution of their symptoms at home.

## Conclusions

Our research indicates that undergoing surgery for acute appendicitis is still safe and feasible amidst the COVID-19 pandemic, with outcomes similar to those before the pandemic. However, we observed an increase in the number of patients with complicated appendicitis, which may have been influenced by national pandemic guidelines in the United Kingdom. Despite this trend, our findings confirm that surgical management for acute appendicitis is still effective during the pandemic, demonstrating the adaptability of healthcare systems in addressing urgent medical needs even in challenging circumstances.

## References

[1] Lyroudi Lyroudi, K. K., Morina Morina, F. F., & Balomenou, C. (2022 Response of Information Technology Companies in Europe to the World Health Organization’s Announcement of the COVID-19 Pandemic. https://doi.org/10.54820/XOWK9116.

[2] Zanke Zanke, A. A., Thenge Thenge, R. R., & Adhao, V. S. (2021 A Pandemic Declare by World Health Organization: COVID-19. Technological Innovation in Pharmaceutical Research Vol. 11, 47-61. https://doi.org/10.9734/bpi/tipr/v11/3766F.

[3] Sukmanee J, Butchon R, Sarajan MH (2022). Estimating the potential overdiagnosis and overtreatment of acute appendicitis in Thailand using a secondary data analysis of service utilization before, during and after the COVID-19 lockdown policy. PLoS One.

[4] Musa Musa, A. A., Wibowo Wibowo, M. D., & Septarendra, D. (2022 Comparison of acute appendicitis severity in pandemic and non-pandemic periods of COVID- 19: A comparative study. Bali Medical Journal, 11, 609-613. https://doi.org/10.15562/bmj.v11i2.3565.

[5] (2020). Elective surgery cancellations due to the COVID-19 pandemic: global predictive modelling to inform surgical recovery plans. Br J Surg.

[6] Sheath C, Abdelrahman M, MacCormick A, Chan D (2021). Paediatric appendicitis during the COVID-19 pandemic. J Paediatr Child Health.

[7] Tankel J, Keinan A, Blich O (2020). The Decreasing Incidence of Acute Appendicitis During COVID-19: A Retrospective Multi-centre Study. World J Surg.

[8] Kamil AM, Davey MG, Marzouk F (2022). The impact of COVID-19 on emergency surgical presentations in a university teaching hospital. Ir J Med Sci.

[9] Suwanwongse K, Shabarek N (2020). Successful Conservative Management of Acute Appendicitis in a Coronavirus Disease 2019 (COVID-19) Patient. Cureus.

[10] Morita K, Fujiogi M, Michihata N, Matsui H, Fushimi K, Yasunaga H, Fujishiro J (2023). Oral Antibiotics and Organ Space Infection after Appendectomy and Intravenous Antibiotics Therapy for Complicated Appendicitis in Children. Eur J Pediatr Surg.

[11] Ganesh R, Lucocq J, Ekpete NO (2020). Management of appendicitis during COVID-19 pandemic; short-term outcomes. Scott Med J.

[12] Orthopoulos G, Santone E, Izzo F, Tirabassi M, Pérez-Caraballo AM, Corriveau N, Jabbour N (2021). Increasing incidence of complicated appendicitis during COVID-19 pandemic. Am J Surg.

[13] Naqvi R H., Mani A., Rometra S. Role of C-reactive protein in enhancing the diagnosis of acute appendicitis.

[14] Ramu Ramu, A. H., Kenchetty Kenchetty, P. P., & Chidananda, A. K. (2021 C-reactive protein, as a marker for predicting acute appendicitis and its severity in KVG Medical College and Hospital, Sullia. International Surgery Journal.

[15] Lotan R, Prosso I, Klatzkin L, Hershkovich O (2022). The Covid 19 Pandemic Effect on the Epidemiology of Thoracolumbar Fractures Presenting to the Emergency Department in Patients Above 65 years Old. Geriatr Orthop Surg Rehabil.

[16] Fisher ND, Bi AS, Aggarwal V, Leucht P, Tejwani NC, McLaurin TM (2021). A Level 1 Trauma Center's response to the COVID-19 pandemic in New York City: a qualitative and quantitative story. Eur J Orthop Surg Traumatol.

[17] De Simone B, Chouillard E, Di Saverio S (2020). Emergency surgery during the COVID-19 pandemic: what you need to know for practice. Ann R Coll Surg Engl.

[18] Waldman R., Kaplan H., & Leitman I M. (2023 Were surgical outcomes for acute appendicitis impacted by the COVID-19 pandemic?. BMC Surgery.

[19] Scheijmans JC, Borgstein AB, Puylaert CA (2021). Impact of the COVID-19 pandemic on incidence and severity of acute appendicitis: a comparison between 2019 and 2020. BMC Emerg Med.

[20] Burgard M, Cherbanyk F, Nassiopoulos K, Malekzadeh S, Pugin F, Egger B (2021). An effect of the COVID-19 pandemic: Significantly more complicated appendicitis due to delayed presentation of patients!. PLoS One.

[21] Zhou Y, Cen LS (2020). Managing acute appendicitis during the COVID-19 pandemic in Jiaxing, China. World J Clin Cases.

[22] Ho S-L, Jun L, Wang C-T, Cheung S-L, Wong K-F, Leung S-K (2021). Impact of Coronavirus 2019 (COVID ‐19) on acute appendicitis in Hong Kong: Retrospective cohort study in a local cluster hospital. Surg Pract.

